# The Role of Tumor Microenvironment in Genomic Instability of Malignant Tumors

**DOI:** 10.3389/fgene.2019.01063

**Published:** 2019-10-29

**Authors:** F. Gizem Sonugür, Hakan Akbulut

**Affiliations:** ^1^Department of Medical Oncology, Ankara University School of Medicine, Ankara, Turkey; ^2^Department of Basic Oncology, Ankara University Cancer Research Institute, Ankara, Turkey

**Keywords:** genomic instability, tumor microenvironment, bone marrow, multiple myeloma, cancer

## Abstract

Genomic instability is an essential feature of cancer cells. The somatic mutation theory suggests that along with inherited ones, the changes in DNA caused by environmental factors may cause cancer. Although approximately 50–60 mutations per tumor are observed in established cancer tissue, it is known that not all of these mutations occur at the beginning of carcinogenesis but also occur later in the disease progression. The high frequency of somatic mutations referring to genomic instability contributes to the intratumoral genetic heterogeneity and treatment resistance. The contribution of the tumor microenvironment to the mutations observed following the acquirement of essential malignant characteristics of a cancer cell is one of the topics that have been extensively investigated in recent years. The frequency of mutations in hematologic tumors is generally less than solid tumors. Although it is a hematologic tumor, multiple myeloma is more similar to solid tumors in terms of the high number of chromosomal abnormalities and genetic heterogeneity. In multiple myeloma, bone marrow microenvironment also plays a role in genomic instability that occurs in the very early stages of the disease. In this review, we will briefly summarize the role of the tumor microenvironment and bone marrow microenvironment in the genomic instability seen in solid tumors and multiple myeloma.

## Genomic Instability in Cancer

### Genomic Instability Is a Hallmark of Cancer Cells

Genomic integrity of cells is maintained through regulated DNA replication, DNA damage repair mechanisms, and cell-cycle checkpoints. The majority of checkpoints in the cells are evolutionally conserved. However, genomic instability itself greatly helped in the diversification of the species throughout the evolutionary process. Genomic instability also plays a significant role in immunoglobulin diversification as well as pathological disorders such as premature aging, some forms of inherited diseases, and cancer (Aguilera and García-Muse, 2013). Along with inborn errors of replication, endogenous reactive metabolites and environmental factors including carcinogen exposure and gamma rays emitted from earth play a role in DNA damage and contribute to genomic instability. More than 100 DNA repair genes act in different pathways to try to maintain the genomic integrity against the factors as mentioned above (Waters, 2006).

Cancer is known as a genetic disease since there has been a genetic selection at the level of single cells having favorable mutations for survival and proliferation. Likewise, many somatic mutations occur in the majority of cancer. Approximately, 40–60 mutations per tumor occur in the majority of solid tumors (Stratton et al., 2009; Bailey et al., 2018). Increases in mutation rate or genomic instability of the tissues are parallel to an increase in the frequency of cancer. The accumulation of genetic and epigenetic alterations in normal tissues has been linked to cancer risk (Takeshima and Ushijima, 2019). Likewise, aneuploidy, a significant indicator of genomic instability, is a common characteristic of cancer and premalignant lesions. A recent analysis from the Mitelman Database revealed that all cancers were aneuploid (Schulze and Petersen, 2011).

Genomic instability is a fundamentally important feature of (all) cancer cells. There are mainly four types of genomic instability: chromosomal instability, intrachromosomal instability, microsatellite instability, and epigenetic instability, described in malignant tumors (Negrini et al., 2010). However, the question of whether the instability is the cause or the consequence of cancer remains unclear. Although the somatic mutation theory suggests cancer as the consequence of the mutations, conditions within the tumor microenvironment (TME) can also induce significant genetic changes in tumor cells. In their seminal work, Reynolds et al. have examined the role of TME on mutagenesis of LNI2 cells (Reynolds et al., 1996). They found that LNI2 cells from mouse tumor explants displayed an increased rate of mutations compared to cells grown in cell cultures under standard conditions. They have also shown that hypoxic conditions in cell culture may induce mutagenesis (Reynolds et al., 1996). Their results indicate that the conditions within solid tumors are mutagenic, which might be a fundamental mechanism of tumor progression. TME could be one of the significant players inducing genomic instability in tumor cells (Chan et al., 2009; Bizzarri and Cucina, 2014).

### Tumor Microenvironment Is an Important Player for Tumor Progression

The healthy tissue microenvironment mainly participates in the maintaining of tissue homeostasis with the cellular interactions and the continuous exchange of factors released by different cellular compartments. However, the TME significantly differs from the healthy tissue microenvironment with its cellular compositions and conditions. The TME is composed of different cell types including cancer cells, cancer-associated fibroblasts (CAFs), endothelial cells, pericytes, macrophages, T lymphocytes, natural killer (NK) cells, mesenchymal stem cells (MSCs), myeloid-derived suppressor cells (MDSCs), and the extracellular matrix (ECM) (Hanahan and Weinberg, 2011). The tumor cells and the other cells mentioned above constitute a unique microenvironment favoring maintaining the malignant properties of the cancer cells. There is a widely accepted link between inflammation, carcinogenesis, and tumor progression. The inflammatory cell subsets, including macrophages, fibroblasts, neutrophils, basophils, and other cells, have cross talk with tumor cells (D'Anselmi et al., 2013). The cross talk between the TME and tumor cells might be related to the differential expression of genes in the reactive tumor stroma. Large-scale gene expression analysis of prostate tumors showed almost 500 genes upregulated, and 600 downregulated, that are mostly cancer-associated pathways (Dakhova et al., 2009). The reactive oxygen species (ROS) produced by the inflammatory cells in the TME may, in turn, induce genetic instability (Radisky et al., 2005). Oxidative DNA damage might occur not only in tumor cells but also in stromal cells. The TME changes caused by oxidative stress may contribute to tumor development and even tumor spreading (Toullec et al., 2010; Jezierska-Drutel et al., 2013).

The TME is mainly characterized by hypoxia, low pH, and nutrient deprivation compared to normal tissue microenvironment. Eukaryotic cells need adequate nutrient supply for optimal mRNA translation. Nutrient deprivation has been shown to inhibit global protein synthesis through modulation of mTOR (Wullschleger et al., 2006) and stress response pathways (Holcik and Sonenberg, 2005). Nutrient deprivation in the TME may induce pro-inflammatory gene expression in cancer cells and further supports tumor progression (Gameiro and Struhl, 2018). Due to poor vascularization and uncontrolled proliferation, cancer cells suffer from nutrient shortage in the TME, which leads to abnormal activation of growth signals (Efeyan et al., 2015).

The healthy tissues usually have O_2_ tension in the range of 20–40 Torr; however, the pO_2_ values of solid tumors could be as low as 1 Torr or less (McKeown, 2014). Decreased glucose and other nutrients and increased levels of toxic metabolites are also characteristic features of common solid tumors (Anastasiou, 2017). Extracellular pH within solid tumors have been shown with values as low as 5.8–6.5, compared to typical values of 7.2–7.4 in well-perfused tissues (Webb et al., 2011). Tumor cells exposed to low pH at 6.5 show significant induction of DNA damage response genes such as EGR1-4 and ATF3 and cell-cycle control genes such as GADD34, GADD45, and p57 (Duggan et al., 2005). Along with hypoxia, low pH induces insulin-like growth factor 1 receptor (IGF1R) expression, which promotes malignant transformation (Peretz et al., 2002).

#### Hypoxia Is a Major Factor Leading to Genomic Instability in TME

Hypoxia has been suggested as the major environmental factor leading to genetic instability of solid tumors. Hypoxia was found to be related to a variety of DNA damage lesions. The first association between hypoxia and DNA damage came from reperfusion injury studies. In reperfusion injury, the most severe tissue damage occurs following the restart of blood flow. The primary mechanism underlying the reperfusion injury is the increase in ROS (Granger and Kvietys, 2015). The increased oxidative stress leads to numerous types of base damage. The most common alterations in purines and pyrimidines are the formation of 8-oxoguanine (8-oxoG) and thymine glycols (Lee et al., 2017). The 8-oxoG can pair with either cytosine or adenine and leads to GC-to-TA transversions (Nakabeppu, 2014).

The potential mechanism seen in reperfusion injury in ischemic tissues is highly relevant to the solid tumors. There is transient and heterogenous hypoxia occurring within the TME. Tumor blood flow is not constant, and substantial blood flow changes have been reported in xenograft tumor models (Aquino-Parsons and Duran, 2001).

The irregular and chaotic nature of the microvessels within the tumors are thought to be the cause of fluctuations in tumor blood supply and oxygen (Braun et al., 1999; Brurberg et al., 2004). Therefore, the fluctuations in red cell flux in tumor vessels may lead to transient hypoxia and reoxygenation cycles in TME. Therefore, these frequent reperfusion cycles within the tumor might induce substantial DNA damage in the TME. During the hypoxia period, ATR kinase causes p53 phosphorylation, and upon reoxygenation, the activated ATM further increases p53 phosphorylation (Hammond et al., 2002), which suggests that the DNA damage mainly occurs during the reoxygenation period following the transient hypoxia in the TME.

ROS is known to induce single- and double-strand breaks of DNA (SSBs and DSBs) in cells. DSBs might increase the rate of translocations, deletions, and gene amplifications seen in most of the cancer cells (Degtyareva et al., 2013; Sharma et al., 2016). The oxygen-dependent nature of the deoxynucleotide supply of the DNA replication during the S-phase is also another source of genomic instability in the TME (Abbas et al., 2013; Turgeon et al., 2018). The shortage of deoxynucleotide precursors during the S-phase may cause a temporary arrest in the S-phase during hypoxia (Ferguson et al., 2015). Likewise, hypoxia has also been shown to cause G1-phase arrest in p53-deficient prostate carcinoma cells *via* induction of p21cip1 and p27kip1, but not in p53 wild-type tumor cells such as MCF7 breast cancer cells and RKO colon cancer cells when exposed to ionizing radiation (Graeber et al., 1994; Gardner et al., 2001).

Hypoxia may induce mutagenesis, especially at the level of small-scale mutations. Reynolds et al. have shown that transient ischemia caused increased mutation rates in cells compared to normoxic ones (Reynolds et al., 1996). The hypoxia-induced mutation rates are usually increased in tumor cells with impaired DNA repair mechanisms. Sharzad et al. have shown increased K-*ras*^G13D^ mutation rates in microsatellite instable colon cancer cells exposed to hypoxia but not in microsatellite stable ones (Shahrzad et al., 2005).

Increasing the hypoxia–reoxygenation cycles further increased the number of mutations. Mutations were common as point mutations and small deletions/insertions (Reynolds et al., 1996).

Hypoperfusion state in the TME causes a significant decrease in the pH level, yielding an acidotic milieu along with decreased levels of glucose and amino acids (Morin et al., 2014). Although lactate is generally considered a waste product, Sonveaux et al. have shown that it is a prominent substrate fueling the oxidative metabolism during the oxygenation of tumor cells in a mouse model of lung carcinoma (Sonveaux et al., 2008).

Likewise, decreased nutrient status in the microenvironment may result in an increased mutation rate. Reynolds et al. have reported a five-times-increased mutation rate of *Escherichia coli* gpt reporter gene in CHO cells when cultured in serum concentrations below 0.25% (Reynolds et al., 1996). However, that increased mutation rate might be related to the increased ROS levels in the culture media, as the addition of anti-oxidants suppressed the mutation rate in their experiments.

DNA mutations may also result in defective repair mechanisms of the cell. DNA mismatch errors might also arise from replication errors which are repaired by mismatch repair (MMR) pathways consisting of a series of proteins including MSH2, MSH3, MSH6, MLH1, and PMS2. Hypoxia has also been shown to induce downregulation of MLH1 and PMS2 both in normal cells and in a variety of cancer cells, including hepatocellular, breast, and colon cancer cells (Nakamura et al., 2008; Rodríguez-Jiménez et al., 2008); while the downregulation of MLH1 occurs at the transcription level, that of PMS2 occurs at the protein level probably through destabilization of the protein (Mihaylova et al., 2003).

**Figure 1:**
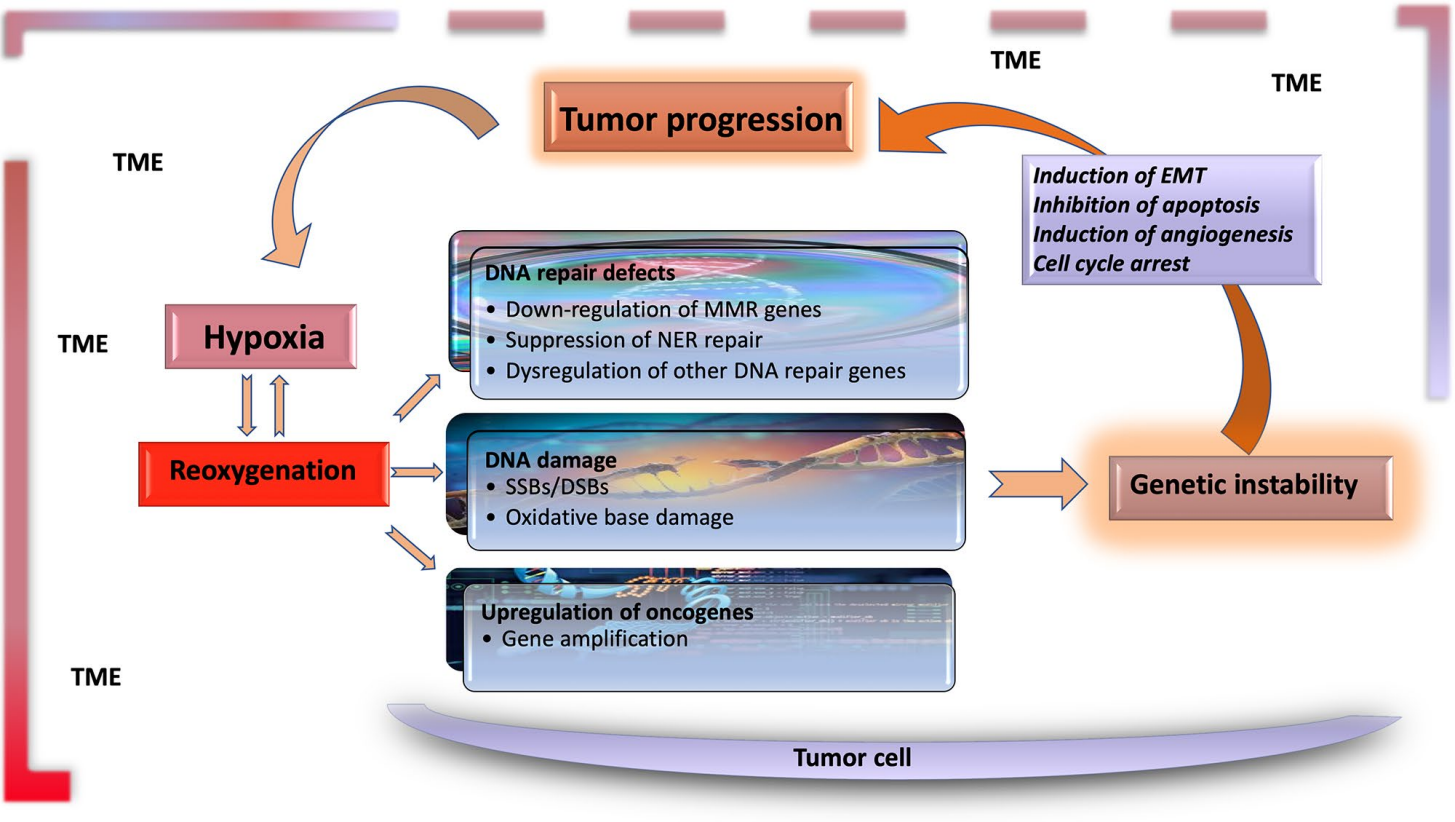
Hypoxia is the major contributor to genomic instability in the tumor microenvironment. Along with inherited changes and the effect of environmental carcinogens, the factors resulting from tumor microenvironment contribute to genomic instability during tumor progression. DNA changes caused by hypoxia–reoxygenation cycles in the tumor microenvironment are an essential source of genomic instability. Functional genetic changes caused by these mutations lead to inhibition of apoptosis, and induction of angiogenesis and EMT, leading to further progression of tumor tissue and the carcinogenic microenvironment. TME, tumor microenvironment; EMT, epithelial–mesenchymal transition; MMR, mismatch repair; NER, nuclear excision repair; SSB, single-strand break; DSB, double-strand break.

The significant characteristics of the TME develop early in solid tumors. In a genetically engineered mouse model, coevolution of the mammary carcinoma cells and their underlying stroma, especially the cancer-associated fibroblasts, has been shown to support most hallmarks of cancer progression and even determine the molecular subtype of breast cancer (Roswall et al., 2018). Likewise, microenvironment-induced instability may contribute to the further development of the tumor. Hypoxia may affect the other DNA repair pathways along with MMR and NER, and this leads to genomic instability in tumors. Hypoxia may also upregulate the genes related to invasion and metastasis. The critical conditions of the TME determining the fate of tumor progression are as follows: massive cell death that results in the release of proteins and additional molecules, hypoxia, low pH level, low glucose levels, shortage of essential nutrients, and abnormal properties of surrounding cells. Hypoxia in the TME mainly results from an imbalance between the oxygen supply and consumption rate. Though the hypoxia is the result of the fast growth rate in tumor tissues, it may further favor tumor progression (Hielscher and Gerecht, 2015). The significant events promoting tumor progression, which are induced by tumor hypoxia, are outlined in **Table 1** and **Figure 1**.

**Table 1:** The major events led by hypoxia in tumor microenvironment.

Mechanisms	Events
Generation of oxygen free radicals	DNA damage, genomic instability
Proteomic and genomic changes including hypoxia-inducible factor I (HIF1)	Adaptation of the tumor cells to hypoxic condition
Activation of genes that are associated with tumor progression	Metabolic adaptation and cell survival
Upregulation of pro-angiogenic pathways	Stimulation of angiogenesis
Inhibition of apoptosis	Tumor mass increase and treatment resistance
Induction of epithelial–mesenchymal transition in tumor cells	Promotion of invasion and metastasis
Downregulation of adhesion molecules	Tumor cell detachment

## Multiple Myeloma, a Disease Between Solid Tumors and Blood Cancers

### Genomic Instability in Multiple Myeloma

Multiple myeloma (MM) is a clonal B-cell malignancy characterized by a significant genetic heterogeneity of different clones that occurred at early stages (Debes-Marun et al., 2003; Munshi and Avet-Loiseau, 2011). Chromosomal abnormalities have long been recognized in patients with MM and plasma cell leukemia. The rate of clones with chromosomal abnormalities can be found to be as much as 35% at the time of diagnosis of MM, 60% in aggressive patients, and 85% in patients with plasma cell leukemia (Calasanz et al., 1997; Avet-Loiseau et al., 2001). Patients with chromosomal abnormalities were found to have features of aggressive disease compared to ones without abnormalities (Calasanz et al., 1997; Difilippantonio et al., 2002).

Genomic instability is a prominent feature of MM. The major genetic abnormalities seen in MM are chromosomal instability, point mutations, and microsatellite instability. MM is more similar to solid tumors than hematologic tumors in terms of increased mutation frequency. Although the role of each mutation is not well understood, the most frequently observed changes are aneuploidy, loss of chromosome 13, and specific translocations involving chromosome 14q32 (Chesi et al., 1997; Facon et al., 2001; Boyd et al., 2012).

The mechanisms that mediate genomic instability and clonal evolution are not well known in MM. It is well known that the chromosomal translocations in the switch region of the immunoglobulin heavy chain (IgH) gene (chromosome 14q32) are usually related to more aggressive disease (Rajan and Rajkumar, 2015). CD40L, IL-6, and IL-4 are known as the critical growth factors for MM cells (Cao et al., 2010; Kamińska et al., 2016). Hwang et al. have shown that CD40 and or IL-4 activation of MM cells induces DNA double-strand breaks and leads to genomic instability (Hwang et al., 2006). Likewise, dysfunctional homologous recombination has also been reported to mediate genomic instability in MM (Shammas et al., 2009). Nuclease activity in the tumor cells produces free ends of DNA, and this may lead to genomic rearrangements. Shammas et al. have shown 34 nucleases whose elevated expression correlated with increased genomic instability in MM (Shammas et al., 2015). Along with homologous recombination (HR), especially, apurinic/apyrimidinic endonuclease activities have been suggested as significant mechanisms for genomic instability (Kumar et al., 2018).

Though the non-silent mutations affecting 13 genes in NER pathway have been reported in some patients with MM, the exact role of this repair mechanism in the generation of genomic instability in MM is not clear (Szalat et al., 2018).

The MYC oncogene activation has been reported to be as much as over 50% of MM patients, which might be related to genomic instability in these patients (Affer et al., 2014). Cottini et al. have shown that MYC regulates ROS levels *via* modulating the activity of mitochondria (Cottini et al., 2015). They also showed that the MYC-induced oxidative stress triggers DNA damage in MM cells.

### Bone Marrow Microenvironment as a Source of Genomic Instability in MM

Although clonal heterogeneity and genomic instability are the significant characteristics of MM even at the earliest stages, DNA damage continues during the disease progression (Neri and Bahlis, 2013). Bone marrow microenvironment has an essential role in the growth and progression of MM.

MM cells are dependent upon the bone marrow microenvironment as hematopoietic stem cells. However, the behavior of myeloma cells greatly differs from the stem cells. Contrary to the stem cell niche, IL-6 is the major contributor for myeloma cells in the bone marrow (Zipori, 2010). IL-6 and other cytokines constitute an inflammatory milieu in the BM for myeloma cells (Zipori, 2010). The increased number of abnormal cells like myeloma cells and leukemia cells in the bone marrow increases angiogenesis. Also, the nature of the bone marrow neovasculature in myeloma resembles the vessels in solid tumors (Tenreiro et al., 2017). Likewise, recent studies have shown that there is a heterogenous O_2_ distribution in bone marrow, and the different parts of the BM have different oxygen tensions (Hu et al., 2010). Therefore, the BM microenvironment could contribute the acquired genomic instability during the disease progression period just as TME of solid tumors.

*In vitro* studies had shown that the DNA breaks already present in tumor cells further increased when the MM cell lines were cocultured with bone marrow stromal cells (Koduru et al., 2012; Neri and Bahlis, 2013; Perini et al., 2017). Koduru et al. have shown that the interaction between MM cells and BM dendritic cells induced the genomic mutator activation-induced cytosine deaminase (AID) and AID-dependent DSBs (Koduru et al., 2012). They also reported that the interaction between DCs and MM cells might further be inhibited by blockade of RANK/RANKL interactions. Coculture of MM cells with BMSc also increased the HR and overall nuclease activity, with a noticeable increase in APEX1, a major apurinic/apyrimidinic nuclease (Perini et al., 2017). Though the exact mechanisms remain unclear, those findings support the hypothesis that bone marrow microenvironment enhances genomic instability in MM.

Tumor mutation burden and presence of neoantigens are usually positively correlated with each other, and an above-average mutation or neoantigen burden was a significant prognostic factor associated with increased risk of disease progression. Likewise, Miller et al. have reported an increased tumor mutation burden in MM patients (Miller et al., 2017). Given that the tumor mutation burden in solid tumors is a useful marker for immune checkpoint inhibitors, it may create a targeted and personalized treatment opportunity in patients with MM.

## Author Contributions

All the authors wrote the manuscript and read and approved the final version of the review.

## Conflict of Interest

The authors declare that the research was conducted in the absence of any commercial or financial relationships that could be construed as a potential conflict of interest.
